# Computational prediction and experimental validation of evolutionarily conserved microRNA target genes in bilaterian animals

**DOI:** 10.1186/1471-2164-11-101

**Published:** 2010-02-09

**Authors:** Kahori Takane, Kosuke Fujishima, Yuka Watanabe, Asako Sato, Nobuto Saito, Masaru Tomita, Akio Kanai

**Affiliations:** 1 Institute for Advanced Biosciences, Keio University, Tsuruoka 997-0017, Japan; 2 Systems Biology Program, Graduate School of Media and Governance, Keio University, Fujisawa 252-8520, Japan

## Abstract

**Background:**

In many eukaryotes, microRNAs (miRNAs) bind to complementary sites in the 3'-untranslated regions (3'-UTRs) of target messenger RNAs (mRNAs) and regulate their expression at the stage of translation. Recent studies have revealed that many miRNAs are evolutionarily conserved; however, the evolution of their target genes has yet to be systematically characterized. We sought to elucidate a set of conserved miRNA/target-gene pairs and to analyse the mechanism underlying miRNA-mediated gene regulation in the early stage of bilaterian evolution.

**Results:**

Initially, we extracted five evolutionarily conserved miRNAs (*let-7*, *miR-1*, *miR-124*, *miR-125/lin-4*, and *miR-34*) among five diverse bilaterian animals. Subsequently, we designed a procedure to predict evolutionarily conserved miRNA/target-gene pairs by introducing orthologous gene information. As a result, we extracted 31 orthologous miRNA/target-gene pairs that were conserved among at least four diverse bilaterian animals; the prediction set showed prominent enrichment of orthologous miRNA/target-gene pairs that were verified experimentally. Approximately 84% of the target genes were regulated by three miRNAs (*let-7, miR-1*, and *miR-124*) and their function was classified mainly into the following categories: development, muscle formation, cell adhesion, and gene regulation. We used a reporter gene assay to experimentally verify the downregulation of six candidate pairs (out of six tested pairs) in HeLa cells.

**Conclusions:**

The application of our new method enables the identification of 31 miRNA/target-gene pairs that were expected to have been regulated from the era of the common bilaterian ancestor. The downregulation of all six candidate pairs suggests that orthologous information contributed to the elucidation of the primordial set of genes that has been regulated by miRNAs; it was also an efficient tool for the elimination of false positives from the predicted candidates. In conclusion, our study identified potentially important miRNA-target pairs that were evolutionarily conserved throughout diverse bilaterian animals and that may provide new insights into early-stage miRNA functions.

## Background

MicroRNAs (miRNAs) are a class of short (18-25 nucleotides) non-coding RNAs that regulate gene expression posttranscriptionally. Their regulatory potential relies heavily on the recognition of binding sites that are located mainly in the 3'-untranslated regions (3'-UTRs) of target messenger RNAs (mRNAs) [1]. Currently, numerous miRNAs with diverse sequences are being characterized in a wide range of species [2], suggesting that this small RNA molecule has a major effect on phylogeny. The importance of miRNAs is also suggested from recent research demonstrating that miRNA-guided gene regulation is involved in diverse biological functions, such as cell differentiation, development, carcinogenesis, and tumour suppression [3-6]. For example, phylogenetically conserved miRNAs (e.g., *let-7*, *miR-1*, *miR-124*, and *miR-125*) are involved in cell differentiation and development [7-10]. In this case, *let-7 *regulates the expression of *RAS *proteins known as critical oncogene products [11]. Moreover, *miR-34*, another evolutionarily conserved miRNA, is a direct downregulator of p53 and is involved in a genetic pathway that promotes cell-cycle progression [12].

	In recent years, more than 700 miRNAs have been identified in humans [13], and this number is increasing. In a recent report by Friedman et al., the expression of a large number of target genes is predicted to be regulated by miRNAs [14]; however, relatively few of these have been verified experimentally. To overcome this problem, a series of computational methods has been developed to predict a large number of miRNA targets; e.g., TargetScan [14], RNAhybrid [15], MicroTar [16], PITA [17], miRanda [18], and PicTar [19]. Nevertheless, these computational approaches often provide numerous target candidates with a large number of false positives because of the weak complementarity between miRNAs and 3'-UTRs [20]. Recently, a phylogenetic profiling approach has been applied to overcome this limitation. For example, studies of the evolution of orthologous target sites have provided insights into the prediction of efficient miRNA targets [21]. As for miRNAs, many miRNA families are found among various bilaterian animals, suggesting that several miRNAs and their target genes may have co-evolved; however, these features have yet to be systematically characterized.

In this study, we hypothesized that the core regulatory relationship between miRNAs and their target genes were conserved throughout the evolution of bilaterian animals. In addition, by predicting these relationships, we sought to elucidate the core function of miRNAs in the primitive gene-regulatory network of the common bilaterian ancestor. Accordingly, we focused on five miRNAs (*let-7*, *miR-1*, *miR-124*, *miR-125/lin-4*, and *miR-34*) that are conserved among bilaterian species (*Homo sapiens *(*H. sapiens*), *Mus musculus *(*M. musculus*), *Gallus gallus *(*G. gallus*), *Drosophila melanogaster *(*D. melanogaster*), and *Caenorhabditis elegans *(*C. elegans*)) and designed a procedure to extract conserved miRNA/target-gene pairs. We extracted evolutionarily conserved miRNA/target-gene pairs based on hybridization patterns and orthologous information. In addition, we experimentally verified several candidate pairs to support our methodology. Our results suggest a functional role of three major miRNAs (*let-7*, *miR-1*, and *miR-124*) that regulated genes related to development, muscle formation, and cell adhesion. These results suggest a new role for the core function of miRNAs in the primitive gene-regulatory network of the common bilaterian ancestor.

## Results and Discussion

### Extraction of evolutionarily conserved miRNAs among five bilaterian animals

To extract conserved miRNA/target-gene pairs, we chose five model species (*H. sapiens*, *M. musculus*, *G. gallus*, *D. melanogaster*, and *C. elegans*) among bilaterian animals, for which there exists a vast array of data on both miRNAs and mRNAs [13]. Previously, several important features were described to classify miRNAs into families. It is well known that the seed sequence (the 5' side of the miRNA sequence) is important for interaction with the target mRNAs [22]. Many miRNA target prediction software programs were developed using the features of seed sequences [14-19]. Moreover, several features have been proposed to identify conserved miRNA families, such as conservation of the mature miRNA sequence (features of the earliest miRNA classification in miRBase) [23] and information on the phylogenetic relationship among miRNAs [24]. By focusing on these features, we proposed the following criteria for extracting well-conserved miRNA families among five species: (1) complete seed sequence matching, (2) mature miRNA sequence identity exceeding 75%, and (3) high conservation among miRNA families, considering the phylogenetic relationship among miRNAs (category I) [24]. Consequently, from 2,404 mature miRNA sequences, we extracted five miRNA families (*let-7, miR-1*, *miR-124*, *miR-125/lin-4*, and *miR-34*) conserved evolutionarily among the five bilaterian animals (Table 1). The sequence identity among most of the conserved miRNA families was over 80%. In particular, the sequences of *let-7 *and *miR-1 *family members showed very high mature miRNA sequence identity (exceeding 90%) among all five bilaterian species, which suggests that a strong selective pressure exists for the nucleotide sequence. Huang et al. described the extraction of 15 conserved miRNA families among six bilaterian animals (*H. sapiens*, *M. musculus*, *G. gallus*, *D. rerio*, *D. melanogaster*, and *C. elegans*) based on their original classification method [24], which included our five miRNA families. In this manuscript, we devised more stringent criteria based on nucleotide conservation to extract highly conserved miRNA families. *Nematostella vectensis *and *Amphimedon queenslandica*, which diverged before the emergence of bilaterian animals, reportedly express various types of miRNAs [25]; however, none of these miRNAs are sequentially similar to the five evolutionarily conserved miRNA families found in the current study (data not shown), which suggests that these evolutionarily conserved miRNAs appeared after the divergence of bilaterian animals or were lost in *N. vectensis *and *A. queenslandica*.

**Table 1:** List of miRNAs conserved among various bilaterian animals

Species						
	
miRNA family	***H. sapiens***	***M. musculus***	***G. gallus***	***D. melanogaster***	***C. elegans***	Identity (%)
***let-7***	*let-7a*	*let-7a*	*let-7a*	*let-7*	*let-7*	95.5
***miR-1***	*miR-1*	*miR-1*	*miR-1a*	*miR-1*	*miR-1*	90.9
***miR-124***	*miR-124*	*miR-124*	*miR-124a*	*miR-124*	*miR-124*	82.6
***miR-125/lin-4***	*miR-125b*	*miR-125b-5p*	*miR-125b*	*miR-125*	*lin-4*	81.8
***miR-34***	*miR-34a*	*miR-34a*	*miR-34a*	*miR-34*	*miR-34*	79.2

### Filtering and enrichment of the evolutionarily conserved miRNA/target-gene pairs

**Figure 1:**
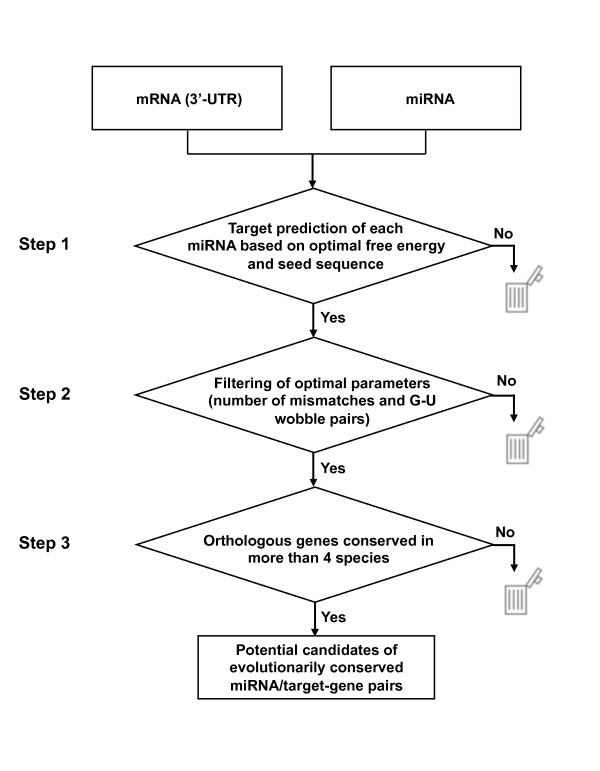
**Computational extraction of conserved miRNA/target gene pairs among bilaterian animals**. Evolutionarily conserved miRNAs were extracted from the five model species (*H. sapiens*, *M. musculus*, *G. gallus*, *D. melanogaster*, and *C. elegans*). For each miRNA, potential target genes were predicted using the following criteria: optimal free-energy threshold and complete matching of nucleotide sequences between the seed sequence of miRNA/mRNA duplexes (step 1), binding pattern of the 3'-UTR of miRNA/mRNA duplexes (step 2), and orthologous gene information (step 3).

To extract genes targeted through evolution by the five conserved miRNAs, we designed a procedure that comprised three screening steps (Figure 1 and Table 2). In step 1, we extracted potential target genes based on optimal free-energy information with a requirement of complete seed-sequence-matching using the RNAhybrid software [15], which predicts potential binding sites of short RNAs among target sequences. For this purpose, the optimal free energy was determined beforehand to efficiently cover experimentally validated miRNA/target-gene pairs. We calculated the free energy of 139 experimentally validated miRNA/target-gene pairs with a complete match of seed sequence, as assessed using RNAhybrid; subsequently, we defined the optimal free energy as < -17 kcal/mole of potential miRNA/target-gene pairs (data not shown). The evaluation of target-gene extraction during each step was carried out using an index termed "Enrichment" (see Methods section). As a result, in step 1, the number of potential target-gene candidates decreased from 357,430 to 153,387, with an enrichment index of 1.8. This prediction set contained 112 of the 145 experimentally validated target-gene pairs.

**Table 2:** Summary of target gene extraction after each screening step

	***H. sapiens***	***M. musculus***	***G. gallus***	***D. melanogaster***	***C. elegans***	Ratio of predicted miRNA/target-gene pairs (%)	Ratio of experimentally verified miRNA/target-gene pairs (%)	Enrichment
**All possible miRNA/gene pairs**	97820	98790	53995	52850	53975	357430/357430 (100)	145/145 (100)	1.0
**STEP 1 (Free energy and seed region)**	56666	53420	20410	14098	8793	153387/357430 (42.9)	112/145 (77.2)	1.8
**STEP 2 (Optimal parameter)**	12405	11256	2524	1118	898	28201/357430 (7.9)	76/145 (52.4)	6.6
**STEP 3**			31			31/10356* (0.3)	4/52 (7.7)	25.7

The total number of possible target genes for the five miRNAs (*let-7*, *miR-1, miR-124, miR-125/lin-4*, and *miR-34*) is represented for the five model species at each screening step (see Figure 1). Asterisks indicate the number of orthologous genes conserved among at least four species. Enrichment was calculated as the "ratio of experimentally verified miRNA/target-gene pairs" divided by the "ratio of predicted miRNA/target-gene pairs".

**Figure 2:**
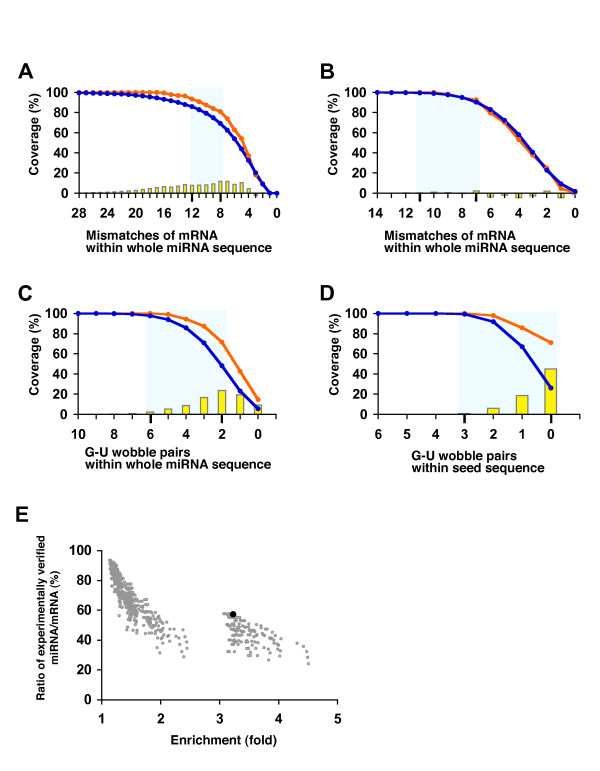
**Parameters used for the prediction of miRNA/mRNA pairs and their coverage**. To optimize the binding parameters of miRNA/mRNA duplexes, we determined the coverage of four binding parameters (mismatch of mRNA within the whole miRNA sequence (A), mismatch of miRNA within the whole miRNA sequence (B), G-U wobble pairs within the whole miRNA sequence (C), and G-U wobble pairs within the seed sequence (D)). Calculation of the coverage was performed using 112 experimentally verified miRNA/mRNA pairs (orange line) and 153,387 predicted miRNA/mRNA pairs (blue line). The yellow bar indicates differences in coverage between experimentally verified and computationally predicted miRNA/mRNA pairs. Four or five points chosen from the highest yellow bar were used as the range of each of the four binding parameters (blue squares) used in this study. We determined parameter space using the binding patterns of the miRNA/mRNA pairs based on four features (E). Five hundred parameter combinations were plotted on a 2D graph using "ratio of experimentally verified miRNA/mRNA" on the Y-axis and "Enrichment" on the X-axis. Black circles (57.3% of the coverage and 3.2-fold of the Enrichment) indicate the point that corresponded to optimized parameters for the prediction of final conserved miRNA/target pairs: 12 for the number of mismatches in the mRNA, 10 for the number of mismatches in the miRNA, 4 for the number of G-U wobble pairs within the whole miRNA sequence, and 0 for the number of G-U wobble pairs within the seed sequence (see Methods section).

In step 2, we considered the 3'-UTR binding pattern of the miRNA/target mRNA. We defined four binding parameters (i.e., number of mismatches of mRNA within the whole miRNA sequence, number of mismatches of miRNA within the whole miRNA sequence, number of G-U wobble pairs within the whole miRNA sequence, and number of G-U wobble pairs within the seed sequence) of the hybridization pattern (Additional file 1) for optimisation of the thresholds for each of the features used to predict reliable miRNA/mRNA pairs. The ranges of the four binding parameters were determined by calculating the coverage of 112 miRNA-mRNA pairs verified experimentally and of 153,387 miRNA-mRNA pairs predicted for each binding feature (Figure 2A-D). Five hundred parameter combinations were plotted on a 2D graph, using "Enrichment" on the X-axis and "Ratio of experimentally verified miRNA/mRNA" on the Y-axis (Figure 2E). From these parameter combinations, we defined the optimal combination of binding parameters for efficient screening based on a maximum EC value of 184.7 (number of mismatches of mRNA within the whole miRNA sequence: 12; number of mismatches of miRNA within the whole miRNA sequence: 10; number of G-U wobble pairs within the whole miRNA sequence: 4; and number of G-U wobble pairs within the seed sequence: 0). Accordingly, the number of potential target-gene candidates was reduced from 153,387 to 28,201 and the experimentally validated target genes decreased from 112 to 76 after introduction of the criterion of optimal hybridization pattern (Enrichment index, 6.6) (Table 2).

Finally, in step 3, we incorporated orthologous gene information and extracted genes that were evolutionarily conserved among more than four diverse bilaterian animals, including *H. sapiens*. As a result, the number of predicted miRNA/target-gene pairs was minimized substantially, from 10,356 to 31, using a significantly high Enrichment index of 25.7 (Table 2). The number of predicted miRNA/target-gene pairs was especially high for the three miRNAs *let-7 *(eight targets), *miR-1 *(seven targets), and *miR-124 *(eleven targets) compared with *miR-125/lin-4 *(three targets) and *miR-34 *(two targets) (Additional file 2). This suggests that *let-7*, *miR-1*, and *miR-124 *may have played a major role in primordial miRNA gene regulation in the common bilaterian ancestor. To verify the significance of conserved miRNA-target gene pairs, we performed same sequence analysis (from step 1 to step 3) against total 25 species-specific miRNAs (5 miRNAs each from 5 species) as a control experiment supposing that these miRNAs are also conserved in other bilaterians. For example, target prediction of hsa-miR-2277, a species-specific miRNA in human was performed in all 5 species (step 1 and step 2) and conserved targets were extracted (step 3). As a result, 11 out of 25 non-conserved miRNAs did not show any conserved miRNA-target gene pair. Furthermore, average number of the miRNA-target gene pairs of the negative control was 2.4, which is statistically lower than that of conserved miRNA-target gene pairs 6.2 based on the Welch's t-test (P < 0.05). These results support that number of genes achieved from the prediction of conserved miRNAs target genes in this study is indeed significant. In summary, we developed a new filtering method for extracting evolutionarily conserved miRNA/target-gene pairs, which was used to extract 31 reliable miRNA/target-gene pairs among the five families of miRNAs.

**Figure 3:**
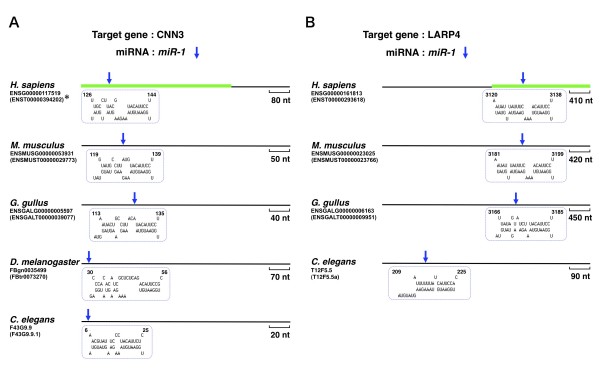
**Two examples of miRNA target sites in orthologous gene transcripts**. Potential target sites of *miR-1 *(blue arrows) in the 3'-UTR sequences of the orthologous *CNN3 *(A) and *LARP4 *(B) transcripts are shown. Predicted duplexes formed by the 3'-UTR sequences (top) and miRNAs (bottom) are shown in dotted boxes for each potential target site. The green bar on the *H. sapiens *3'-UTR sequence indicates a DNA region used for the construction of the reporter plasmid pLuc-CNN3 (Figure 4A). See Additional file 3 for other candidates. (*) The length of the CNN3 3'-UTR is currently registered as a little shorter than that indicated (527 nt in size) and contains the *miR-1 *binding site (Ensembl release 53).

We discovered that only one orthologous target gene, calponin-3 (*CNN3*), was conserved completely among the five bilaterian animals (Figure 3A). As for target genes conserved in four species, we found, for example, the La-related protein 4 (*LARP4*), ETS domain-containing protein Elk-3 (*ELK3*), argonaute-4 (*EIF2C4*), transgelin-2 (*TAGLN2*), and V-type proton ATPase subunit B brain isoform (*ATP6V1B2*) genes (Figure 3B and Additional file 3). Of note, the same approximate position of the predicted target site was observed in the orthologous 3'-UTR of *CNN3 *(120 nucleotides (nt)), *LARP4 *(3,100 nt), *EIF2C4 *(220 nt), and *TAGLN2 *(50 nt) among vertebrates (Figure 3 and Additional file 3). According to Bartel et al., the distribution of miRNA target sites within the 3'-UTR is biased near the mRNA stop codon or poly-A tail compared with the middle portion of 3'-UTR [26]. Our results show that target site distribution varied according to the type of miRNA target gene. The target sites on 3'-UTR of *CNN3 *and *TAGLN2 *were biased near the stop codon, from *H. sapiens *to *C. elegans *(Figure 3A and Additional file 3). Regarding the other candidates, we observed all types of target site distribution on 3'-UTR. A future statistical analysis of miRNA target-site distribution among conserved miRNA/target-gene pairs is required to substantiate this view. With the exception of 3'-UTR of the *LARP4 *gene, most of the binding patterns of evolutionarily conserved target sites were sequentially different, without taking the seed region into consideration. The target-site binding patterns within 3'-UTR of the *LARP4 *gene were identical between *H. sapiens *and *M. musculus *(Figure 3B), although the similarity of the two 3'-UTR sequences was ~70% (data not shown). A recent study reported on cooperative regulation by multiple miRNAs [19]. Likewise, the band 4.1-like protein 4B (*EPB41L4B*) gene was an orthologous target of two different types of miRNAs: *miR-1 *and *miR-124 *(Table 3). Our analysis suggests that multiple miRNA regulation may have already existed in the era of ancestral bilaterian species.

**Table 3:** Evolutionarily conserved genes regulated by miRNAs

Target gene							
					
ID	Name	Function	***let-7***	***miR-1***	***miR-124***	***miR-125/lin4***	***miR-34***
**Candidates targeted by multiple miRNAs**							

ENSG00000095203	EPB41L4B	Band 4.1-like protein 4B		+**	**+**		

**Candidates targeted by single miRNAs**							

ENSG00000187772	LIN28B	Lin-28 homolog B	**+**				
ENSG00000198799	LRIG2	Leucine-rich repeats and immunoglobulin-like domains protein 2 precursor	**+**				
ENSG00000086544	ITPKC	Inositol-trisphosphate 3-kinase C	**+**				
ENSG00000196233	LCOR	Ligand-dependent corepressor	**+**				
ENSG00000139263	LRIG3	Leucine-rich repeats and immunoglobulin-like domains protein 3 precursor	**+**				
ENSG00000134698	EIF2C4	Eukaryotic translation initiation factor 2C 4	**+**				
ENSG00000170456	DENND5B	MGC24039 protein	**+**				
ENSG00000136231	IGF2BP3	Insulin-like growth factor 2 mRNA-binding protein 3	**+**				
ENSG00000158710	TAGLN2	Transgelin-2		+*			
ENSG00000143549	TPM3	Tropomyosin alpha-3 chain		+*			
ENSG00000117519	CNN3	Calponin-3		**+**			
ENSG00000071073	MGAT4A	Alpha-1,3-mannosyl-glycoprotein 4-beta-N-acetylglucosaminyltransferase A		**+**			
ENSG00000161813	LARP4	La-related protein 4		**+**			
ENSG00000147416	ATP6V1B2	Vacuolar ATP synthase subunit B, brain isoform		**+**			
ENSG00000135862	LAMC1	Laminin subunit gamma-1 precursor			+*		
ENSG00000125695	STRADA	STE20-related adapter protein			**+**		
ENSG00000131459	GFPT2	Glucosamine--fructose-6-phosphate aminotransferase			**+**		
ENSG00000111145	ELK3	ETS domain-containing protein Elk-3			**+**		
ENSG00000151726	ACSL1	Long-chain-fatty-acid--CoA ligase 1			**+**		
ENSG00000164144	ARFIP1	Arfaptin-1			+**		
ENSG00000080819	CPOX	Coproporphyrinogen III oxidase, mitochondrial precursor			**+**		
ENSG00000138279	ANXA7	Annexin A7			**+**		
ENSG00000150093	ITGB1	Integrin beta-1 precursor			+**		
ENSG00000093167	LRRFIP2	Leucine-rich repeat flightless-interacting protein 2			**+**		
ENSG00000116141	MARK1	Serine/threonine-protein kinase MARK1				**+**	
ENSG00000166797	FAM96A	Protein FAM96A				**+**	
ENSG00000131914	LIN28	Lin-28 homolog A				+*	
ENSG00000113282	CLINT1	EPN4_HUMAN Isoform 2 of Q14677 - Homo sapiens					**+**
ENSG00000137872	SEMA6D	Semaphorin-6D precursor					**+**

List of conserved targets regulated by conserved miRNAs. Transcript of Band 4.1-like protein 4B is only regulated by two miRNAs (*miR-1 *and *miR-124*). "*" and "**" indicates experimentally verified miRNA-mRNA and possible miRNA-mRNA candidates revealed by microarray data, respectively.

**Figure 4:**
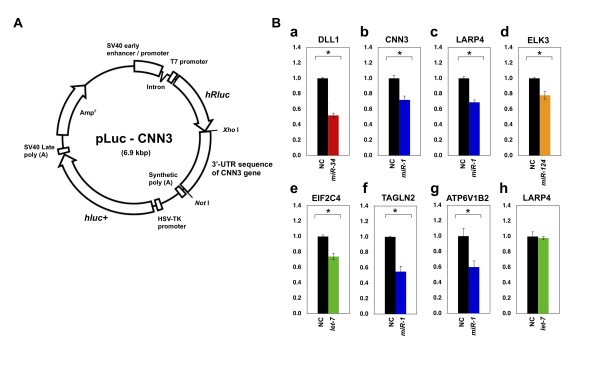
**Example of the 3'-UTR reporter plasmid and experimental validation**. The 3'-UTR sequences of *DLL1, CNN3, LARP4, ELK3, EIF2C4, TAGLN2*, and *ATP6V1B2 *were subcloned into the *Xho*I/*Not*I site of the psiCHECK™-2 vector. *CNN3 *was chosen as representative of the eight candidates listed above (see Methods section). (B) HeLa cells were cotransfected with each combination of 100 ng of reporter plasmid and the indicated amounts of each miRNA (*DLL1*, 5 pmol of *miR-34 *(a); *CNN3*, 60 pmol of *miR-1 *(b); *LARP4*, 20 pmol of *miR-1 *(c); *ELK3*, 60 pmol of *miR-124 *(d); *EIF2C4*, 60 pmol of *let-7 *(e), *TAGLN2*, 5 pmol of *miR-1 *(f), *ATP6V1V2*, 60 pmol of *miR-1 *(g), and *LARP4*, 60 pmol of *let-7 *(h)). Colours depict each miRNA: *miR-34 *(red), *miR-1 *(blue), *miR-124 *(orange), *let-7 *(green), and negative control (black). The relative expression of the luciferase gene was measured 24 h after transfection. The normalized luciferase activity of the control vector was set as 1.0. The data represent the average of three experiments and SDs. * *P *< 0.01.

### Experimental validation of miRNA target genes

To validate the evolutionarily conserved miRNA/target-gene candidates, we performed transfection and luciferase assays on 6 of the 31 identified evolutionarily conserved miRNA/target-gene candidates. Four of the six candidates (*CNN3, LARP4, TAGLN2*, and *ATP6V1B2*) were predicted to be regulated by *miR-1*, while one candidate (*ELK3*) was predicted to be targeted by *miR-124*, and the final candidate (*EIF2C4*) was predicted to be the target of *let-7*. We used the well-established downregulation of the delta-like protein 1 precursor (*DLL1*) gene by *miR-34 *as a positive control [22], and the *LARP4*/*let-7 *pair, which was extracted up to step 2 in our analysis, was chosen as a non-evolutionarily conserved pair. We subcloned the 3'-UTR sequence downstream from the *Renilla *luciferase gene (Figure 4A) and co-transfected 100 ng of the 3'-UTR reporter construct into HeLa cells using 5, 20, and 60 pmol of miRNA Mimics (hsa-let-7a, hsa-miR-1, hsa-miR-124, hsa-miR-34a, and miRIDIAN microRNA Hairpin Inhibitor Negative Control #1) (see Methods section). We observed the downregulation of six out of six candidates and of the positive control compared with the negative controls (Figure 4B, a-g). Typical results of the reporter gene assay are shown in Figure 4B for the indicated amounts of miRNAs (5, 20, and 60 pmol). The downregulation of these candidates was significant (*P *< 0.01), although some of these pairs represented an inhibition of only 30% under the current conditions. Among these candidates, *TAGLN2 *was previously suggested to be downregulated by *miR-1*, as assessed by microarray analysis [27]. This feature was recently confirmed using the "pulsed stable isotope labelling with amino acids in cell culture" (pSILAC) method and a reporter gene assay [28]. Regarding the *let-7*/*LARP4 *combination, the expression of *LARP4 *was not downregulated after *let-7 *transfection, which was supported statistically (Figure 4B, h). These experimental results suggest that our new method has the potential for efficiently extracting reliable miRNA/target-gene pairs and may be effective in the elucidation of the primordial regulatory relationships between miRNAs and their target genes during the early stage of bilaterian evolution.

### Possible regulation of evolutionarily conserved miRNA targets in bilaterian animals

To provide further insight into the primary functions of evolutionarily conserved miRNAs (Table 3), we next focused on the functions of the target genes and found that evolutionarily conserved miRNA/target genes could be largely classified into four functional categories: development, differentiation, muscle movement, and gene regulation. First, we describe the function of evolutionarily conserved genes involved in development and differentiation. The laminin subunit gamma-1 precursor (*LAMC1*) gene, which was possibly regulated by *miR-124*, is one of the major components of the basement membrane. According to Smyth et al. [29], null mutation of *LAMC1 *causes embryonic lethality because of the absence of the basement membrane and failure to differentiate the endoderm. Among other candidates regulated by *miR-124*, the expression of the leucine-rich repeat flightless-interacting protein 2 (*LRRFIP2*) gene induces an extra axis in *Xenopus laevis *embryos [30]. Moreover, the semaphorin-6D precursor (*SEMA6D*) gene, plays an important role in cardiac morphogenesis during chick embryonic dev which was predicted as a candidate of *miR-34 *targeting, elopment [31]. The *lin-28 *gene, which regulates developmental timing in *C. elegans*, is reportedly controlled by *lin-4*, as assessed using *in vivo *experiments [32]. Subsequently, the *lin-28 *gene was also found to be regulated by *miR-125*, which is an orthologous miRNA of *lin-4*, in *H. sapiens *and *M. musculus *[33]. Our prediction confirmed the regulation of orthologous *lin-28 *genes by *lin-4/miR-125 *miRNA in *H. sapiens*, *M. musculus*, and *C. elegans *and further suggested that a similar regulatory relationship was conserved in *G. gallus*. Interestingly, our prediction showed that the orthologous *let-7*/*lin-28 *pair was also evolutionarily conserved among bilaterians. This feature had been validated experimentally in *H. sapiens *[34]; however, in *C. elegans*, *lin-28 *is expressed in an early stage of development, while *let-7 *is expressed in a later stage of development, suggesting that regulation of *lin-28 *by *let-7 *is subtle in *C. elegans *[7]. It would be of interest to analyse the interaction of the *let-7*/*lin-28 *pair in other species, such as *M. musculus *and *G. gallus*, to understand whether stage-specific expression of *let-7 *was present in the common ancestor of bilaterian animals or if it is a trait acquired later during evolution, as many of the evolutionarily conserved miRNA/target-gene pairs were related to an essential function involved in differentiation and development.

Next, we focused on the tissue-specific miRNA/target genes. Among the 30 evolutionarily conserved target candidates, approximately one-third were expressed in a tissue-specific manner in humans, according to the BioGPS portal http://biogps.gnf.org. *miR-1 *is highly expressed in muscle tissues [8]. Here, three candidate genes regulated by *miR-1 *(i.e., *TAGLN2*, *CNN3*, and *TPM3*) are also expressed in muscle tissues, according to BioGPS. *TAGLN2 *is a homolog of *TAGLN*, which encodes an actin-binding protein and is a diagnostic marker of breast and colon carcinoma in humans [35]. The *CNN3 *gene also encodes an actin-binding protein that represses bone morphogenetic protein (BMP) signalling in chondrocytes, which is important for bone formation [36]. Moreover, the *TPM3 *gene encodes yet another actin-binding protein that modulates muscle contraction. The other miRNA, *miR-124*, is expressed in the nervous system [9]. Similarly, one of the evolutionarily conserved target candidates, the glucosamine-fructose-6-phosphate aminotransferase 2 (*GFPT2*) gene, is expressed in the central nervous system [37]. In addition, the annexin A7 (*ANXA7*) gene, which is another candidate target of *miR-124*, is involved in the development of the murine brain [38]. The striking overlap between the tissue specificity of evolutionarily conserved miRNA and that of their target genes suggests that one of the main functions of primordial miRNAs may have been the regulation of genes implicated in the temporary control of the development of muscle and of the nervous system, in a tissue-specific manner.

Finally, we found two interesting candidate genes, *EIF2C4 *and *LARP4*, which encode translation-related proteins. It is well accepted that miRNAs are regulators of gene expression, mostly at the translational level [1]. *EIF2C4 *is also known as Argonaute 4 (*AGO4*). Although the function of AGO4 is unknown, other Argonaute protein family members are involved in the RNA-induced silencing complex (RISC), which is essential for the miRNA or siRNA pathways. A previous microarray analysis performed in HepG2 cells revealed that the *EIF2C4 *gene was affected by *let-7 *[6]. In the present study, we demonstrated for the first time the direct downregulation of *EIF2C4 *by *let-7*, as assessed using a reporter gene assay in HeLa cells (Figure 4B, e); therefore, we speculate that negative-feedback regulation of *EIF2C4 *by *let-7 *exists in the miRNA pathway. It has been reported that regulation of the *AGO1 *mRNA, which is a major component of the RISC complex, in the miRNA pathway by *miR-168 *controls plant development in *Arabidopsis thaliana *[39]. Another candidate, *LARP4*, encodes a member of La-motif protein family that controls translational efficiency [40]. We also demonstrated the downregulation of the *LARP4 *gene via *miR-1 *using a reporter gene assay (Figure 4B, c), which further supports our contention that some of the evolutionarily conserved miRNAs may play an important role in the regulation of translation by controlling the expression levels of translation factors and by negatively regulating their own miRNA pathway.

## Conclusions

We developed a procedure to extract potential evolutionarily conserved miRNA/target-gene pairs based on orthologous gene information from five bilaterian animals, and efficiently extracted 31 evolutionarily conserved miRNA/target-gene pairs from 357,430 pairs. We experimentally validated the downregulation of six candidate pairs (out of six tested pairs) in HeLa cells, which suggests that our method using orthologous information was efficient in extracting evolutionarily conserved miRNA target-gene candidates. Our findings reveal that miRNA target sites were conserved among various species, and demonstrate that especially *let-7*-, *miR-1*-, and *miR-124*-mediated gene regulation may have played an important role throughout evolution, in processes such as development, differentiation, and muscle movement. Moreover, our results indicate that miRNA-mediated translational regulation as well as tissue-specific expression of miRNA/target-gene pairs may have already existed in the common bilaterian ancestor. In conclusion, our study will provide new insights into the early stages of miRNA function.

## Methods

### miRNA and 3'-UTR sequence data

We downloaded 2,404 mature miRNA sequences (885 for *H. sapiens*, 689 for *M. musculus*, 520 for *G. gallus*, 153 for *D. melanogaster*, and 157 for *C. elegans*) from the miRBase, version 13.0 http://microrna.sanger.ac.uk/sequences/[13]. We downloaded sequences corresponding to 3'-UTR using the Ensembl transcript ID annotation in FASTA format (40,498 transcripts for *H. sapiens*, 3,332 for *M. musculus*, 13,089 for *G. gallus*, 16,822 for *D. melanogaster*, and 13,560 for *C. elegans*) from the Ensmart database Ensembl release 53 http://www.ensembl.org/index.html[41]. Orthologous gene information was also downloaded from the Ensmart database Ensembl release 53. We obtained 145 experimentally verified miRNA/target-gene pairs from TarBase Version 5.0.1 http://diana.cslab.ece.ntua.gr/tarbase/[42].

### Identification of miRNAs conserved among bilaterian animals

The 2,404 miRNA sequences were aligned using ClustalX [43] with the following alignment parameters: gap opening, 22.50; gap extension, 0.83; and bootstrap value, 100. We checked the conservation of 2,404 miRNA sequences to extract evolutionarily conserved miRNAs. We defined the conservation threshold as an "overall sequence identity > 75% with complete matching of the seed sequence (1-7, 2-8, or 3-9 nucleotides from the miRNA's 5' end)"; furthermore, we introduced information on the phylogenetic relationship among miRNAs to extract reliably conserved miRNAs and used highly conserved miRNA families (category I) [24].

### Extraction of evolutionarily conserved miRNA/target-gene pairs among bilaterian animals

To extract evolutionarily conserved miRNA/target-gene pairs among bilaterian animals, we devised a three-step filtering approach (Figure 1). In step 1, we predicted genes targeted by each of the five miRNAs (*let-7*, *miR-1*, *miR-124*, *miR-125/lin-4*, and *miR-34*) using RNAhybrid, which is fast and flexible software for miRNA target prediction, with the free-energy option and the seed-sequence option [15]. The RNA duplex free-energy filter was defined as the appropriate value that led to the efficient extraction of experimentally verified miRNA/target-gene pairs. We also considered a complete match across the seed sequence (1-7, 2-8, or 3-9 nucleotides from the miRNA's 5' end), which was used as a filter by adding the seed option of RNAhybrid.

In step 2, we used four binding parameters of the hybridization pattern of the miRNA/mRNA duplexes. According to a recent study, a binding rule is likely to exist for the recognition of target mRNAs by miRNAs [34]. Moreover, G-U wobble pairs within miRNA/mRNA duplexes play a key role in the interaction with target mRNAs [44]. Subsequently, potential candidates were extracted using four binding parameters (number of mismatches of mRNA within the whole miRNA sequence, number of mismatches of miRNA within the whole miRNA sequence, number of G-U wobble pairs within the whole miRNA sequence, and number of G-U wobble pairs within the seed sequence) of the miRNA/mRNA duplexes (Additional file 1). We used the hybridization pattern of experimentally verified and predicted miRNA/mRNA pairs to calculate coverage, by changing these binding parameters one by one (28-0 for the number of mismatches of mRNA within the whole miRNA sequence; 14-0 for the number of mismatches of miRNA within the whole miRNA sequence; 10-0 for the number of G-U wobble pairs within the whole miRNA sequence; and 6-0 for the number of G-U wobble pairs within the seed sequence) (Figure 2A-D). The range of each of the four binding parameters was determined based on the coverage of experimentally verified and predicted miRNA/mRNA pairs. Parameter combinations were then plotted on a 2D graph by calculating the "ratio of experimentally verified miRNA/mRNA" and "Enrichment" using the points in the four parameter ranges (Figure 2E). The criterion "Enrichment" was defined and calculated as the value of "ratio of experimentally verified miRNA/target-gene pairs" divided by the "ratio of predicted miRNA/target-gene pairs". We obtained the most effective combination of four binding parameters for extracting miRNA/target-gene pairs based on the EC value. Parameter conbination with highest EC value was selected.

In step 3, orthologous gene information was used to extract orthologous genes targeted by the same type of miRNA. For the retrieval of evolutionarily conserved miRNA/target-gene pairs from various bilaterian animals, we set the orthologous gene information criteria as orthologous genes conserved in at least four species, each containing the miRNA target site of interest.

### Expression vectors

To construct target-site reporter plasmids, each DNA fragment (3'-UTR sequence of the *DLL1 *gene (668 nt; accession no. AF003522), *ELK3 *gene (519 nt; accession no. BC017371), *EIF2C4 *gene (2148 nt; accession no. AB046787), *TAGLN2 *gene (1391 nt; accession no. D21261), *LARP4 *gene (1678 nt; accession no. AY004310), *CNN3 *gene (1391 nt; accession no. BC025372), and *ATP6V1B2 *gene (1208 nt; accession no. L35249)) was amplified from HeLa genomic DNA via polymerase chain reaction using site-specific primers and was inserted into the *Xho*I/*Not*I sites of the psiCHECK-2 plasmid vector (which encodes both firefly and *Renilla *luciferases; Promega, Madison, WI, USA) (Figure 4A). The oligonucleotide was designed to introduce *Xho*I and *Not*I sites at the 5' and 3' termini, respectively. The resulting plasmids were termed pLuc-DLL1, pLuc-CNN3, pLuc-LARP4, pLuc-ELK3, pLuc-EIF2C4, pLuc-TAGLN2, and pLuc-ATP6V1B2, respectively. miRIDIAN™ miRNA Mimic for hsa-let-7a, hsa-miR-1, hsa-miR-124, hsa-miR-34a, and negative control (miRIDIAN microRNA Hairpin Inhibitor Negative Control #1) were purchased from Dharmacon. miRNA Mimic molecules are chemically modified double-stranded RNA oligonucleotides.

The sequences of the oligodeoxyribonucleotides used for PCR were as follows:

(1) DLL1_S: 5'-TTACTCGAGAATGGAAGTGAGATGGCAAGAC-3'

(2) DLLl_A: 5'-TTAGCGGCCGCTTGTCATTCATAAAATTTATTT-3'

(3) CNN3_S: 5'-TTACTCGAGTCCACACAGAAGGAGCTCAG-3'

(4) CNN3_A: 5'-TTAGCGGCCGCCAGGAAGAGCAAATGCATCA-3'

(5) LARP4_S: 5'-TTACTCGAGGTTCCCATTTGATGGCATGT-3'

(6) LARP4_A: 5'-TTAGCGGCCGCCATAGCACCTTGGCGATGTT-3'

(7) ELK3_S: 5'-TTACTCGAGCGTCTGGCCACAATTAAGGA-3'

(8) ELK3_A: 5'-TTAGCGGCCGCTGCTTTCATATTGCCCACTG-3'

(9) EIF2C4_S: 5'-TTACTCGAGTTCTACCAGCAGCTCGGAAT-3'

(10) EIF2C4_A: 5'-TTAGCGGCCGCTTGGATTCCAGCAAGTCCTC-3'

(11) TAGLN2_S: 5'-TTACTCGAGCCCTCCCACGAATGGTTAAT-3'

(12) TAGLN2_A: 5'-TTAGCGGCCGCATGGAAAATGAGAAGCCACG-3'

(13) ATP6V1B2_S: 5'-TTACTCGAGTCCGCGCTCTTGTGAAATAC-3'

(14) ATP6V1B2_A: 5'-TTAGCGGCCGCATAATCATGCTGACTCCCCC-3'

### Transfection and luciferase reporter assay

Transient transfection and luciferase assays were performed as described previously, with slight modifications [22]. Briefly, HeLa cells were grown in 10% FBS in DMEM and seeded in 24-well plates 24 h before transfection. Cells were transfected with the indicated amounts of reporter and miRNA Mimic (100 ng of target reporter and 5, 20, and 60 pmol of miRNA Mimic) in the presence of Lipofectamine 2000 (Invitrogen, Carlsbad, CA, USA). Firefly and *Renilla *luciferase activities were measured consecutively using the Dual-luciferase assay system (Promega) 24 h after transfection, according to the manufacturer's instructions.

## List of abbreviations used

miRNA: microRNA; 3'-UTR: 3'-untranslated region; EC value: multiplied value of Enrichment and Coverage; nt: nucleotides; SD: standard deviation.

## Authors' contributions

KT participated in all aspects of the study. KF contributed to the overall conception of this work and performed the functional profiling of miRNA-targeted genes. YW supported the design of the target-gene extraction procedure and validated new miRNA target candidates using transfection and luciferase reporter assay experiments. AS constructed the experimental system. NS participated in the validation of new miRNA target candidates using transfection and luciferase reporter assay experiments. MT supervised the project. AK participated in the experimental design and drafted the manuscript. All authors read and approved the final manuscript.

## Supplementary Material

Additional file 1 **Basic concept of miRNA/mRNA duplex formation**. An example of the binding pattern of miRNA (bottom)/mRNA (top) duplexes is shown using *cel-let-7 *and die-1 3'-UTR sequences from *C. elegans*. The green and blue squares depict mismatched nucleotide sequences of the mRNA and miRNA, respectively. The red square depicts G-U wobble pairs within the whole miRNA sequence and the black arrow pinpoints a G-U wobble pair within the seed sequence. Click here for file

Additional file 2 Summary of the number of target genes in each extraction step Click here for file

Additional file 3 **Additional examples of miRNA target sites in orthologous gene transcripts used for experimental verification**. Potential target sites of *miR-124 *(orange arrows) in the 3'-UTR sequences of orthologous *ELK3 *transcripts (A). Potential target sites of *let-7 *(green arrows) in the 3'-UTR sequences of orthologous *EIF2C4 *transcripts (B). Potential target sites of *miR-1 *(blue arrows) in the 3'-UTR sequences of orthologous *TAGLN2 *transcripts (C) and *ATP6V1B2 *transcripts (D). 3'-UTR sequences and miRNAs are shown in dotted boxes for each potential target site; the colours of dotted boxes and arrows correspond to those of each miRNA. Click here for file
