# Supplementary material for: Computational prediction and experimental validation of evolutionarily conserved microRNA target genes in bilaterian animals
Source: BMC Genomics. 2010 Feb 9;11:101. doi: 10.1186/1471-2164-11-101 (PMC2833159; doi:10.1186/1471-2164-11-101)

Additional file 1. Basic concept of miRNA/mRNA duplex formation

An example of the binding pattern of miRNA (bottom)/mRNA (top) duplexes is shown using *cel-let-7* and die-1 3′-UTR sequences from *C. elegans*. The green and blue squares depict mismatched nucleotide sequences of the mRNA and miRNA, respectively. The red square depicts G-U wobble pairs within the whole miRNA sequence and the black arrow pinpoints a G-U wobble pair within the seed sequence.

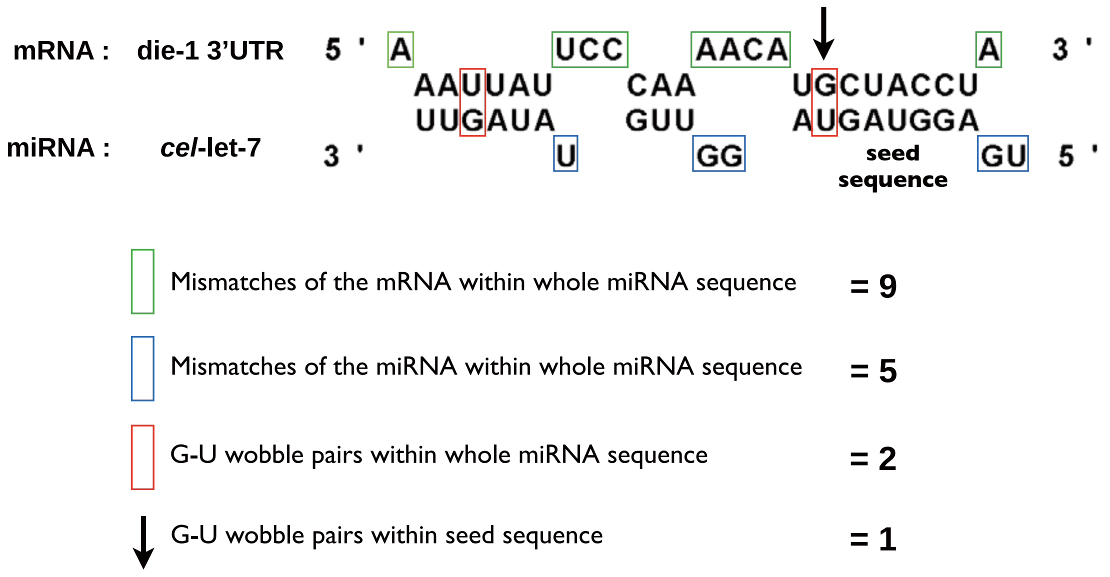

Supplement: Additional file 1 — Basic concept of miRNA/mRNA duplex formation. An example of the binding pattern of miRNA (bottom)/mRNA (top) duplexes is shown using cel-let-7 and die-1 3'-UTR sequences from C. elegans. The green and blue squares depict mismatched nucleotide sequences of the mRNA and miRNA, respectively. The red square depicts G-U wobble pairs within the whole miRNA sequence and the black arrow pinpoints a G-U wobble pair within the seed sequence. [file 1471-2164-11-101-S1.PDF]
