# Supplementary material for: Computational prediction and experimental validation of evolutionarily conserved microRNA target genes in bilaterian animals
Source: BMC Genomics. 2010 Feb 9;11:101. doi: 10.1186/1471-2164-11-101 (PMC2833159; doi:10.1186/1471-2164-11-101)
Supplement: Additional file 2 — Summary of the number of target genes in each extraction step [file 1471-2164-11-101-S2.PDF]

Additional file 2. Summary of the number of target genes in each extraction step

A. *let-7*

	H. sapiens	*M. musculus*	*G. gallus*	*D. melanogaster*	*C. elegans*
Step 1	14099	13522	5353	3168	2416
Step 2	2064	1870	360	207	161
Step 3			8		

B. *miR-1*

	H. sapiens	*M. musculus*	*G. gallus*	*D. melanogaster*	*C. elegans*
Step 1	6958	6006	1811	2658	1547
Step 2	1807	1549	395	378	333
Step 3			7		

C. *miR-124*

	H. sapiens	*M. musculus*	*G. gallus*	*D. melanogaster*	*C. elegans*
Step 1	10813	10534	4248	2247	1611
Step 2	2881	2591	667	222	237
Step 3			11		

D. *miR-125/lin-4*

	H. sapiens	*M. musculus*	*G. gallus*	*D. melanogaster*	*C. elegans*
Step 1	9854	9013	2282	917	408
Step 2	2855	2557	436	82	61
Step 3			3		

E. *miR-34*

	H. sapiens	*M. musculus*	*G. gallus*	*D. melanogaster*	*C. elegans*
Step 1	14942	14345	6716	5108	2811
Step 2	2798	2689	666	229	106
Step 3			2		
