# Supplementary material for: Computational prediction and experimental validation of evolutionarily conserved microRNA target genes in bilaterian animals
Source: BMC Genomics. 2010 Feb 9;11:101. doi: 10.1186/1471-2164-11-101 (PMC2833159; doi:10.1186/1471-2164-11-101)
Supplement: Additional file 3 — Additional examples of miRNA target sites in orthologous gene transcripts used for experimental verification. Potential target sites of miR-124 (orange arrows) in the 3'-UTR sequences of orthologous ELK3 transcripts (A). Potential target sites of let-7 (green arrows) in the 3'-UTR sequences of orthologous EIF2C4 transcripts (B). Potential target sites of miR-1 (blue arrows) in the 3'-UTR sequences of orthologous TAGLN2 transcripts (C) and ATP6V1B2 transcripts (D). 3'-UTR sequences and miRNAs are shown in dotted boxes for each potential target site; the colours of dotted boxes and arrows correspond to those of each miRNA. [file 1471-2164-11-101-S3.PDF]

Additional file 3. Additional examples of miRNA target sites in orthologous gene transcripts used for experimental verification

Potential target sites of *miR-124* (orange arrows) in the 3′-UTR sequences of orthologous *ELK3* transcripts (A). Potential target sites of *let-7* (green arrows) in the 3′-UTR sequences of orthologous *EIF2C4* transcripts (B). Potential target sites of *miR-1* (blue arrows) in the 3′-UTR sequences of orthologous *TAGLN2* transcripts (C) and *ATP6V1B2* transcripts (D). 3′-UTR sequences and miRNAs are shown in dotted boxes for each potential target site; the colours of dotted boxes and arrows correspond to those of each miRNA.

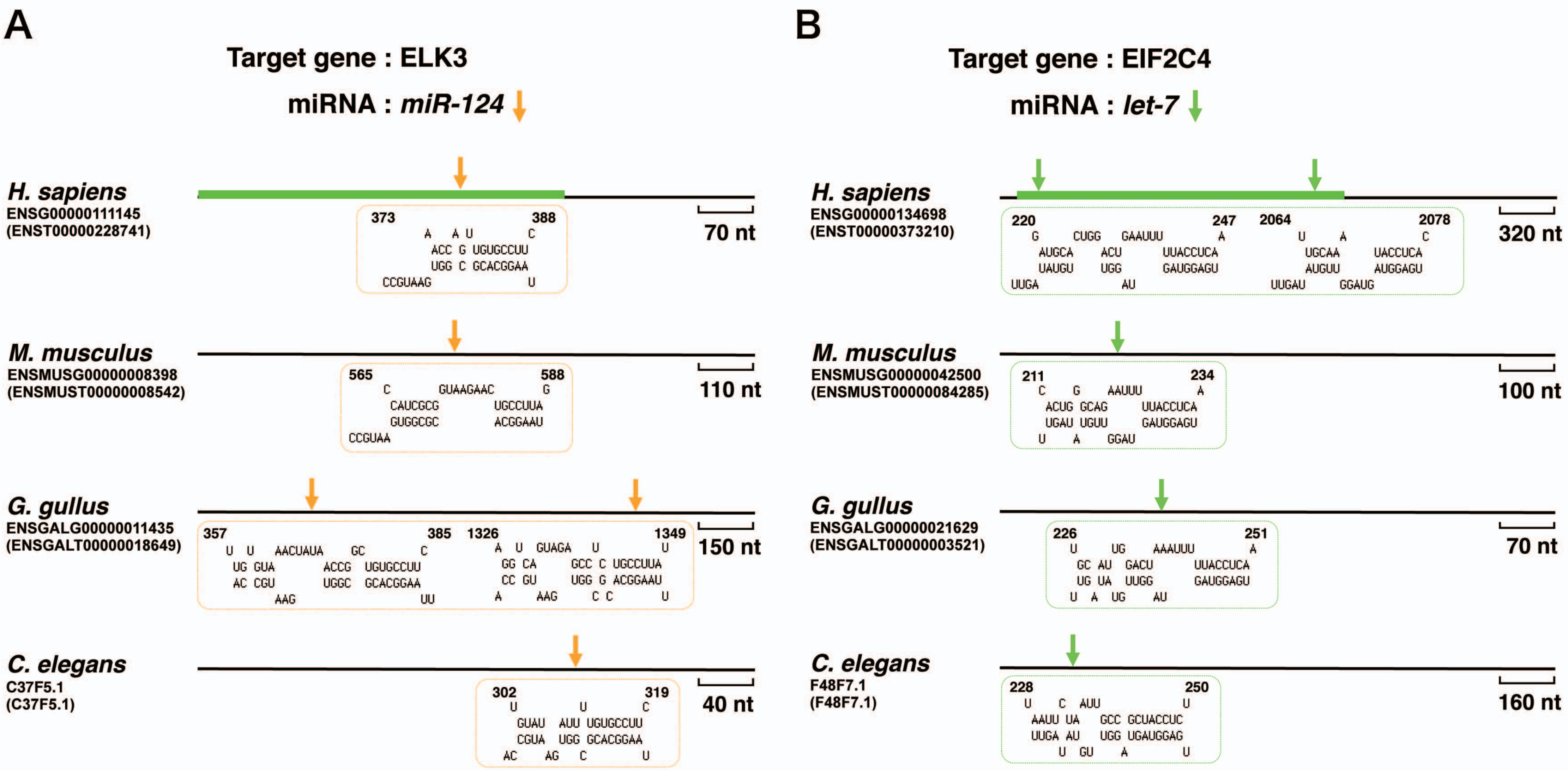
A
Target gene : ELK3
miRNA : miR-124
H. sapiens
ENSG00000111145
(ENST00000228741)
373 388
A A U C
ACC G UGUGCCUU
UGG C GCACGGAA
CCGUAAG U
70 nt
M. musculus
ENSMUSG00000008398
(ENSMUST00000008542)
565 588
C GUAAGAAC G
CAUCGCG UGCCUUA
GUGGCGC ACGGAAU
CCGUAA
110 nt
G. gullus
ENSGALG00000011435
(ENSGALT00000018649)
357 385
U U AACUAUA GC C
UG GUA ACCG UGUGCCUU
AC CGU UGGC GCACGGAA
AAG UU
1326 1349
A U GUAGA U U
GG CA GCC C UGCCUUA
CC GU UGG G ACGGAAU
A AAG C C U
150 nt
C. elegans
C37F5.1
(C37F5.1)
302 319
U U C
GUAU AUU UGUGCCUU
CGUA UGG GCACGGAA
AC AG C U
40 nt
B
Target gene : EIF2C4
miRNA : let-7
H. sapiens
ENSG00000134698
(ENST00000373210)
220 247
G CUGG GAAUUU A
AUGCA ACU UUACCUCA
UAUGU UGG GAUGGAGU
UUGA AU
2064 2078
U A C
UGCAA UACCUCA
AUGUU AUGGAGU
UUGAU GGAUG
320 nt
M. musculus
ENSMUSG00000042500
(ENSMUST00000084285)
211 234
C G AAUUU A
ACUG GCAG UUACCUCA
UGAU UGUU GAUGGAGU
U A GGAU
100 nt
G. gullus
ENSGALG00000021629
(ENSGALT00000003521)
226 251
U UG AAAUUU A
GC AU GACU UUACCUCA
UG UA UUGG GAUGGAGU
U A UG AU
70 nt
C. elegans
F48F7.1
(F48F7.1)
228 250
U C AUU U
AAUU UA GCC GCUACCUC
UUGA AU UGG UGAUGGAG
U GU A U
160 nt

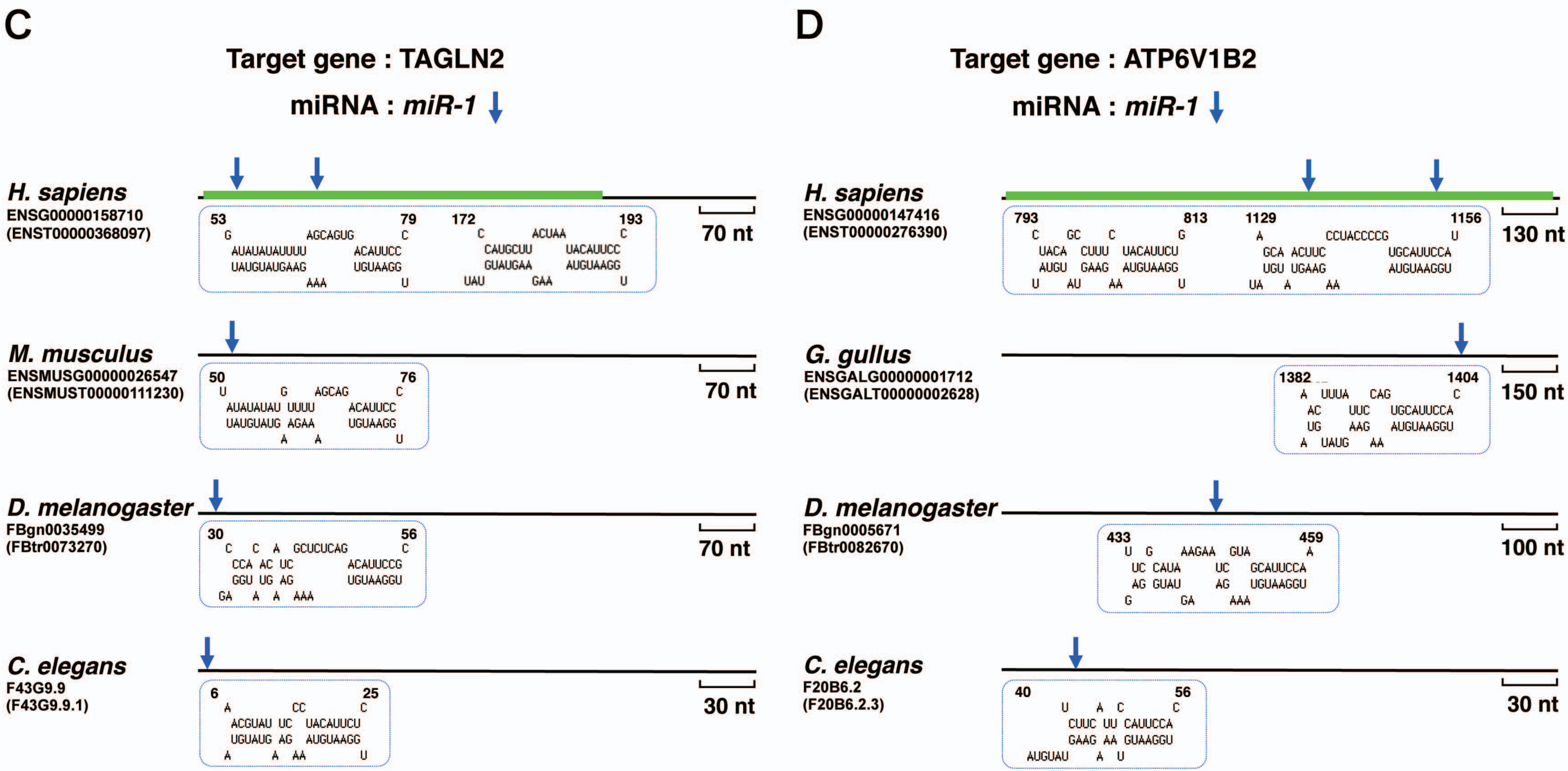
C
Target gene : TAGLN2
miRNA : miR-1
H. sapiens
ENSG00000158710
(ENST00000368097)
53 79 172 193
G AGCAGUG C C ACUAA C
AUAUAUAUUUU ACAUUCC CAUGCUU UACAUUCC
UAUGUAUGAAG UGUAAGG GUAUGAA AUGUAAGG
AAA U UAU GAA U
70 nt
M. musculus
ENSMUSG00000026547
(ENSMUST00000111230)
50 76
U G AGCAG C
AUAUAUAU UUUU ACAUUCC
UAUGUAUG AGAA UGUAAGG
A A U
70 nt
D. melanogaster
FBgn0035499
(FBtr0073270)
30 56
C C A GCUCUCAG C
CCA AC UC ACAUUCCG
GGU UG AG UGUAAGGU
GA A A AAA
70 nt
C. elegans
F43G9.9
(F43G9.9.1)
6 25
A CC C
ACGUAU UC UACAUUCU
UGUAUG AG AUGUAAGG
A A AA U
30 nt
D
Target gene : ATP6V1B2
miRNA : miR-1
H. sapiens
ENSG00000147416
(ENST00000276390)
793 813 1129 1156
C GC C G A CCUACCCCG U
UACA CUUU UACAUUCU GCA ACUUC UGCAUUCCA
AUGU GAAG AUGUAAGG UGU UGAAG AUGUAAGGU
U AU AA U UA A AA
130 nt
G. gullus
ENSGALG00000001712
(ENSGALT00000002628)
1382 1404
A UUUA CAG C
AC UUC UGCAUUCCA
UG AAG AUGUAAGGU
A UAUG AA
150 nt
D. melanogaster
FBgn0005671
(FBtr0082670)
433 459
U G AAGAA GUA A
UC CAUA UC GCAUUCCA
AG GUAU AG UGUAAGGU
G GA AAA
100 nt
C. elegans
F20B6.2
(F20B6.2.3)
40 56
U A C C
CUUC UU CAUUCCA
GAAG AA GUAAGGU
AUGUAU A U
30 nt
